# Acute Intestinal Obstruction Revealing an Internal Hernia of the Broad Ligament Following Promontofixation: A Case Report

**DOI:** 10.7759/cureus.90080

**Published:** 2025-08-14

**Authors:** Lamribah Mohamed, Hamza Mougib, Ayoub M Madani, Mohammed Ouazni, Mehdi Soufi

**Affiliations:** 1 Surgery, Hospital Mohammed VI Agadir, Agadir, MAR; 2 Visceral Surgery, Souss Massa University Hospital Center, Agadir, MAR; 3 General Surgery, Mohammed VI University Hospital, Faculty of Medicine and Pharmacy, Oujda, MAR; 4 General Surgery, Souss Massa University Hospital Center, Faculty of Medicine and Pharmacy, Ibn Zohr University, Agadir, MAR; 5 General Surgery, Souss Massa University Hospital Center, Agadir, MAR

**Keywords:** broad ligament, internal hernia, intestinal obstruction, laparoscopy, promontofixation

## Abstract

Internal hernias through the broad ligament are a rare cause of acute intestinal obstruction, often discovered intraoperatively. We report the case of a 75-year-old woman who presented to the emergency department with abdominal pain evolving over three days, associated with vomiting and obstipation (inability to pass stool and flatus). Contrast-enhanced abdominopelvic CT imaging revealed a mechanical small bowel obstruction with a pelvic transition zone, suggesting either an internal hernia of the broad ligament or a postoperative adhesion. Initial medical management was instituted, given the absence of signs of bowel ischemia. This case highlights the importance of considering this rare condition in the differential diagnosis of unexplained bowel obstruction in women, even in the absence of initial signs of severity.

## Introduction

Internal hernias through the broad ligament are an exceptional cause of intestinal obstruction, accounting for less than 7% of all internal hernias [1]. These hernias involve the passage of a bowel loop through an abnormal orifice in the broad ligament of the uterus. Their diagnosis is often made incidentally during surgical exploration for acute bowel obstruction. In this context, contrast-enhanced abdominopelvic CT is a valuable diagnostic tool [2]. We present a representative case of a 75-year-old woman with a history of laparoscopic promontofixation who presented with acute intestinal obstruction due to a suspected broad ligament hernia.

## Case presentation

The patient, aged 75, had a medical history, including a non-operated hiatal hernia, hemithyroidectomy, cholecystectomy, hysteroscopy for a benign polyp, two vaginal deliveries, and laparoscopic promontofixation for pelvic organ prolapse performed five years earlier, without postoperative complications.

She presented to the emergency department with a three-day history of abdominal pain, vomiting, and obstipation, without fever. On examination, she was hemodynamically stable and afebrile. Her abdomen was soft, with tenderness in the pelvic and epigastric regions, without guarding or rigidity. Per rectal examination revealed an empty rectum.

Laboratory results are summarized in Table 1, highlighting leukocytosis and elevated CRP, with mild liver function abnormalities and a urinary tract infection.

**Table 1:** Laboratory findings on admission

Parameter	Patient value	Normal range
White blood cells (WBC)	24,000 /µL	4,000 – 10,000 /µL
C-reactive protein (CRP)	42 mg/L	< 5 mg/L
Hemoglobin	13.6 g/dL	12 – 16 g/dL (female)
Creatinine	95 µmol/L	45 – 90 µmol/L (female)
Urea	16 mmol/L	2.5 – 7.5 mmol/L
ALT (alanine aminotransferase)	Mildly elevated ×2N	< 35 IU/L
Bilirubin (indirect)	Mild elevation	< 17 µmol/L (total)
Sodium (Na⁺)	Normal	135 – 145 mmol/L
Potassium (K⁺)	Normal	3.5 – 5.0 mmol/L
Venous lactate	0.85 mmol/L	0.5 – 2.2 mmol/L
Urine culture	Escherichia coli	Negative (no growth)

**Figure 1:**
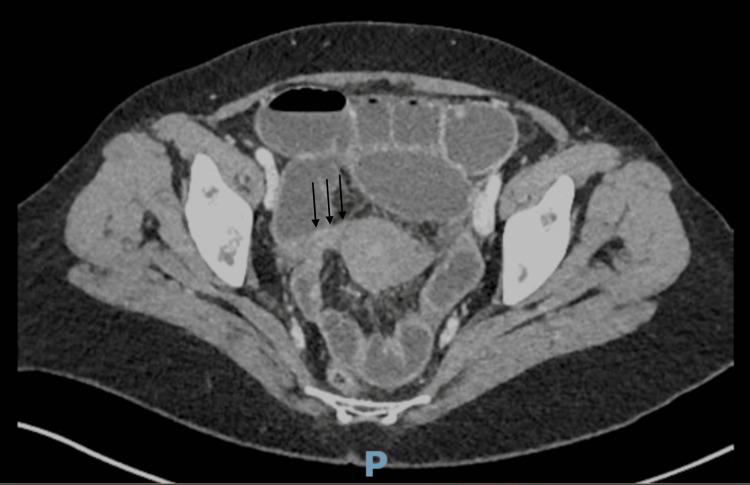
Axial slice of contrast-enhanced abdominopelvic CT showing small bowel distension with a pelvic transition zone suggestive of a broad ligament hernia or postoperative adhesion

Contrast-enhanced abdominopelvic CT revealed mechanical small bowel obstruction with diffuse small bowel distension (maximum diameter ~3.5 cm) and a pelvic transition zone located near the right broad ligament, suggestive of a broad ligament hernia or postoperative adhesion. No bowel wall thickening, mesenteric vessel engorgement, or abnormal bowel wall enhancement was observed, indicating preserved vascularity (Figure 1) [3].

Given the patient’s stable condition and absence of CT signs of strangulation, initial conservative management was attempted. This included intravenous rehydration, correction of electrolyte imbalances, nasogastric decompression, and antibiotics. Despite biochemical improvement, clinical obstruction persisted after 72 hours. A gastrografin challenge was inconclusive, with contrast stasis.

Diagnostic laparoscopy was performed. Exploration revealed diffuse small bowel dilatation with early signs of bowel distress but no ischemia, a right pelvic transition zone, and a small amount of clear peritoneal fluid. The colon was non-dilated. The small bowel was mobilized cephalad, exposing the pelvis.

**Figure 2:**
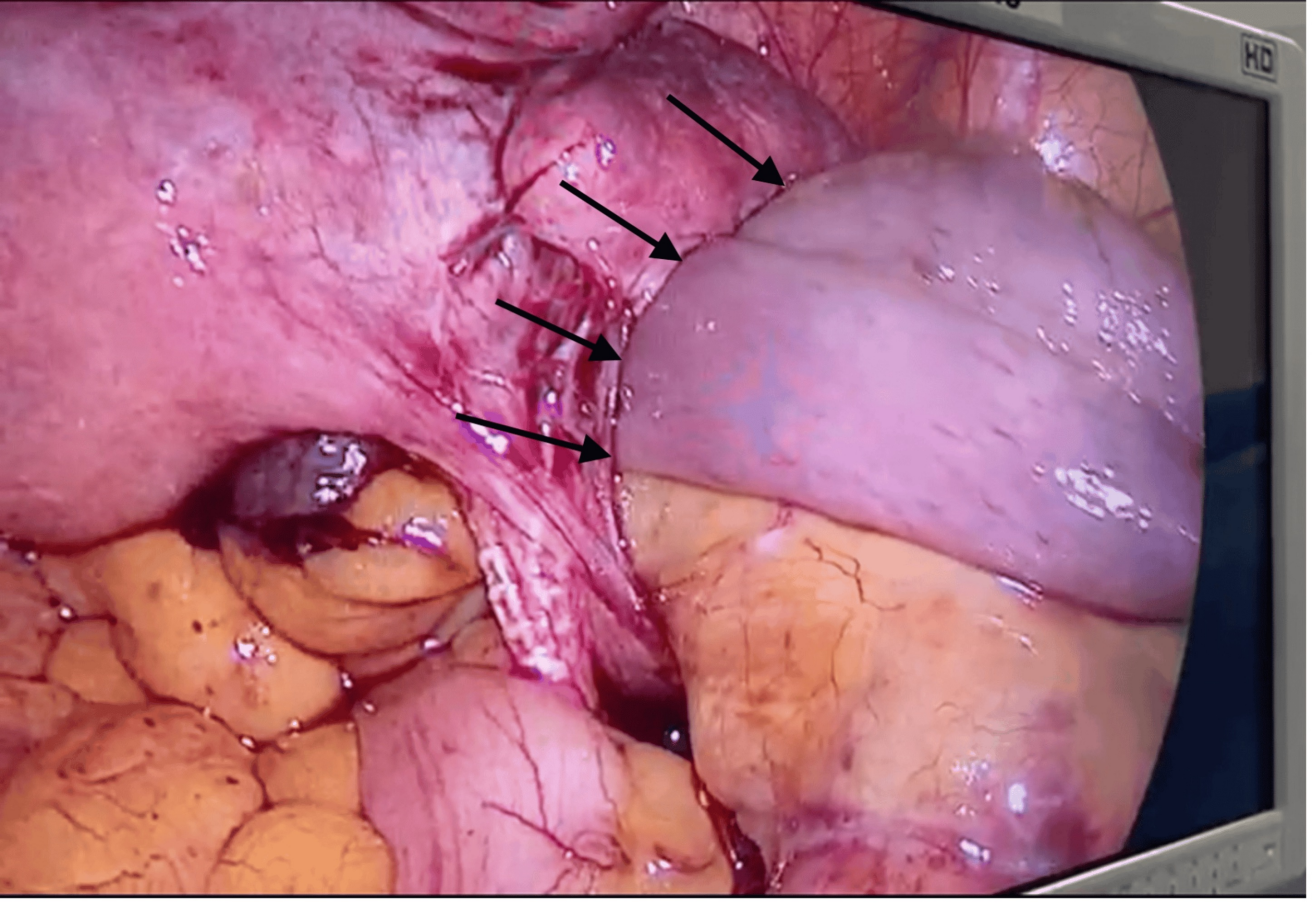
Intraoperative laparoscopic view showing incarceration of the ileal loop through a defect in the right broad ligament

An internal hernia through the right broad ligament was identified, with a 2 cm defect bordered superiorly by the peritoneal fold associated with the promontofixation mesh. The penultimate ileal loop was incarcerated (Figure 2).

**Figure 3:**
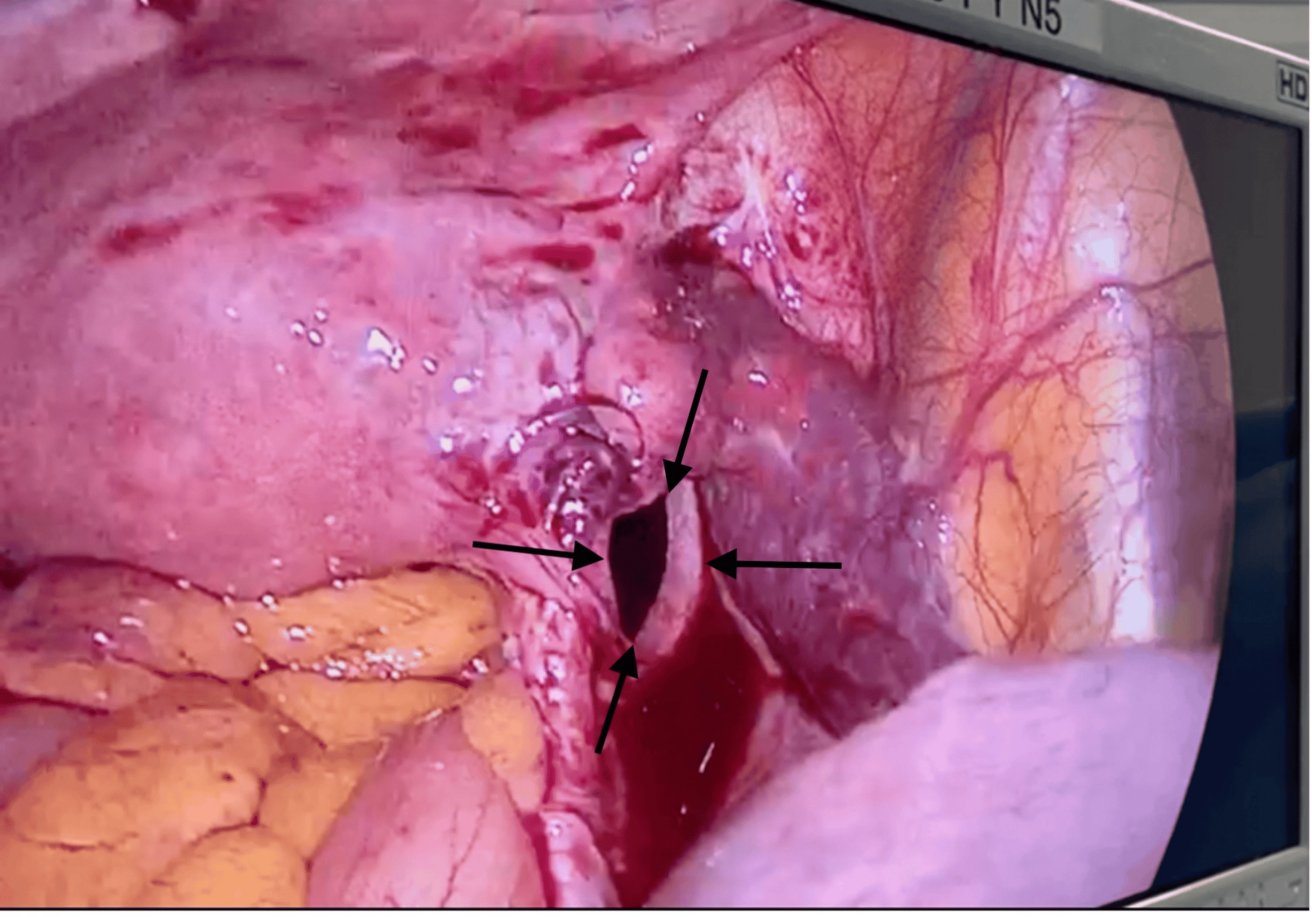
Post-reduction view of the broad ligament hernial orifice

The loop was carefully reduced without injury or ischemia. The hernial orifice was closed with a non-absorbable V-Lock® 2.0 suture, using superficial peritoneal stitches (Figure 3). No defect was found on the left side. Postoperative recovery was uneventful with return of bowel function on postoperative day 2 and discharge on day 3.

## Discussion

Internal hernias of the broad ligament represent an extremely rare but potentially serious cause of small bowel obstruction. First described in the early twentieth century [4], these hernias are classified based on the anatomical location of the defect, most commonly involving the fenestration between the two layers of the broad ligament. The etiology can be congenital, due to developmental anomalies, such as persistence of embryologic structures (e.g., remnants of the Müllerian ducts), or acquired, resulting from trauma, pelvic inflammatory disease, or previous gynecologic surgery [5].

In our case, the presence of a promontofixation mesh fold likely created a pathological orifice, supporting the hypothesis of an acquired origin. This finding is especially relevant considering the increased number of laparoscopic pelvic procedures performed in recent years. Surgeons should be aware of this iatrogenic risk, especially in patients with a history of pelvic reconstructive surgery.

The clinical presentation is typically non-specific and indistinguishable from other causes of mechanical small bowel obstruction. Symptoms may include crampy abdominal pain, nausea, vomiting, abdominal distension, and obstipation. The lack of pathognomonic signs makes diagnosis particularly challenging and often delayed [6].

Imaging, especially with contrast-enhanced CT, plays a critical role in preoperative suspicion. Although signs such as closed-loop obstruction, abnormal location of bowel loops in the pelvis, and displacement of gynecological organs may suggest the diagnosis, definitive identification of the hernial orifice is rarely achieved preoperatively [2]. In our patient, the CT was suggestive but not conclusive.

Laparoscopy offers both diagnostic and therapeutic advantages. It allows direct visualization of the defect and assessment of bowel viability. Moreover, it facilitates gentle manipulation and repair in a minimally invasive fashion, thereby reducing postoperative pain, hospital stay, and recovery time. In our case, the laparoscopic approach enabled a safe and effective reduction of the incarcerated bowel and secure closure of the defect with a non-absorbable suture [3].

Alternative surgical approaches, such as laparotomy, may be indicated in cases of hemodynamic instability, suspected bowel necrosis, or extensive adhesions. However, when feasible, laparoscopy remains the preferred method due to its diagnostic precision and therapeutic efficiency.

Failure to diagnose and treat broad ligament hernias promptly can result in bowel strangulation, ischemia, and necrosis, significantly increasing morbidity and mortality. Thus, early surgical exploration is warranted when conservative measures fail or clinical deterioration is observed.

## Conclusions

Broad ligament internal hernias, though extremely rare, should be considered in the differential diagnosis of small bowel obstruction, especially in women with prior pelvic surgeries such as promontofixation. Preoperative imaging with contrast-enhanced CT can suggest the diagnosis, but laparoscopy remains the most reliable method for confirmation and treatment.

Early recognition and prompt surgical intervention are crucial to avoid complications such as bowel ischemia or necrosis. This case highlights the importance of maintaining a high index of suspicion in atypical cases of bowel obstruction and demonstrates the effectiveness of a minimally invasive approach for both diagnosis and repair. When laparoscopy is not feasible, open surgery remains an alternative, but with potentially longer recovery times.

## References

[1] Lê P, Chambon H, Madeuf E (2005). Hernie interne du ligament large [Article in French]. J Chir (Paris).

[2] Kosaka N, Uematsu H, Kimura H, Yamamori S, Hirano K, Itoh H (2007). Utility of multi-detector CT for pre-operative diagnosis of internal hernia through a defect in the broad ligament (2007: 1b). Eur Radiol.

[3] Marraoui W, Petitcolin V, Bros S, Slim K, Garcier JM, Da Ines D (2012). Internal hernia of the broad ligament: CT diagnosis for laparoscopic management. Diagn Interv Imaging.

[4] Hunt AB (1934). Fenestra and pouches in the broad band ligament as an actual and potential cause of strangulated intraabdominal hernia. Surg Gynecol Obstet.

[5] Cissé M, Ka I, Konaté I, Ka O, Dieng M, Dia A, Touré CT (2011). Incarcerated internal hernia through a breach of the broad ligament, a case report [Article in French]. Gynecol Obstet Fertil.

[6] Ngabou UD, Hornez E, Chiron P, Fondin M, Pons F (2012). Internal hernia through a defect in the broad ligament. J Visc Surg.

